# Discovery of a Novel Pseudo‐Natural Product Aurora Kinase Inhibitor Chemotype through Morphological Profiling

**DOI:** 10.1002/advs.202309202

**Published:** 2024-04-03

**Authors:** Lin Wang, Furkan Yilmaz, Okan Yildirim, Beate Schölermann, Sukdev Bag, Luca Greiner, Axel Pahl, Sonja Sievers, Rebecca Scheel, Carsten Strohmann, Christopher Squire, Daniel J. Foley, Slava Ziegler, Michael Grigalunas, Herbert Waldmann

**Affiliations:** ^1^ Department of Chemical Biology Max Planck Institute of Molecular Physiology 44227 Dortmund Germany; ^2^ Faculty of Chemistry and Chemical Biology TU Dortmund University 44227 Dortmund Germany; ^3^ Compound Management and Screening Center (COMAS) 44227 Dortmund Germany; ^4^ Faculty of Chemistry and Inorganic Chemistry TU Dortmund University 44227 Dortmund Germany; ^5^ School of Biological Sciences University of Auckland 1142 Auckland New Zealand; ^6^ School of Physical and Chemical Sciences University of Canterbury 8041 Christchurch New Zealand

**Keywords:** Aurora kinase inhibitor, indole dearomatization, interrupted fischer indole, morphological profiling, pseudo‐natural products

## Abstract

The pseudo‐natural product (pseudo‐NP) concept aims to combine NP fragments in arrangements that are not accessible through known biosynthetic pathways. The resulting compounds retain the biological relevance of NPs but are not yet linked to bioactivities and may therefore be best evaluated by unbiased screening methods resulting in the identification of unexpected or unprecedented bioactivities. Herein, various NP fragments are combined with a tricyclic core connectivity via interrupted Fischer indole and indole dearomatization reactions to provide a collection of highly three‐dimensional pseudo‐NPs. Target hypothesis generation by morphological profiling via the cell painting assay guides the identification of an unprecedented chemotype for Aurora kinase inhibition with both its relatively highly 3D structure and its physicochemical properties being very different from known inhibitors. Biochemical and cell biological characterization indicate that the phenotype identified by the cell painting assay corresponds to the inhibition of Aurora kinase B.

## Graphical abstract

Various NP fragments are synthetically combined with a tricyclic core connectivity via interrupted Fischer indole and indole dearomatization reactions to provide a collection of highly three‐dimensional pseudo‐natural products. Target hypothesis generation by morphological profiling guides the identification of a truly unprecedented chemotype for Aurora kinase inhibition with both its relatively highly three‐dimentional structure and its physicochemical properties being very different from known inhibitors.

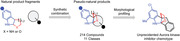

## 1 Introduction

The design of compound collections that can efficiently explore biologically relevant chemical space is a key criterion for successful molecular discovery programs. Such design concepts will be particularly fruitful if they draw from molecular architectures with proven meaningfulness to Nature, and which encode binding to protein targets in their three‐dimensional structure. Natural products (NPs) are the result of Nature's exploration of biologically relevant chemical space through evolution and have been a rich source of therapeutics^[^ ^1^ ^]^ as well as an inspiration for molecular design principles^[^ ^2^ ^]^ including diversity‐oriented synthesis,^[^ ^3^ ^]^ complexity‐to‐diversity,^[^ ^4^ ^]^ and biology‐oriented synthesis.^[^ ^5^ ^]^ Nevertheless, natural evolution is a slow process that has limited Nature's exploration of chemical space to only a fraction of possible NP‐like structures.^[^ ^6^ ^]^ By employing evolutionary logic along with the tools of synthetic chemistry, the pseudo‐NP concept aims to accelerate the evolutionary process of exploring NP‐like chemical space by fusing NP fragments in combinations and/or arrangements that are not found in Nature.^[^ ^7^ ^]^ The novel scaffolds retain the biological relevance of NPs but are not accessible by Nature through known biosynthetic pathways. In conjunction with suitable screening technologies, pseudo‐NP collections may lead to the discovery of novel chemotypes for unexpected targets or the identification of unprecedented bioactivities.

**Figure 1:**
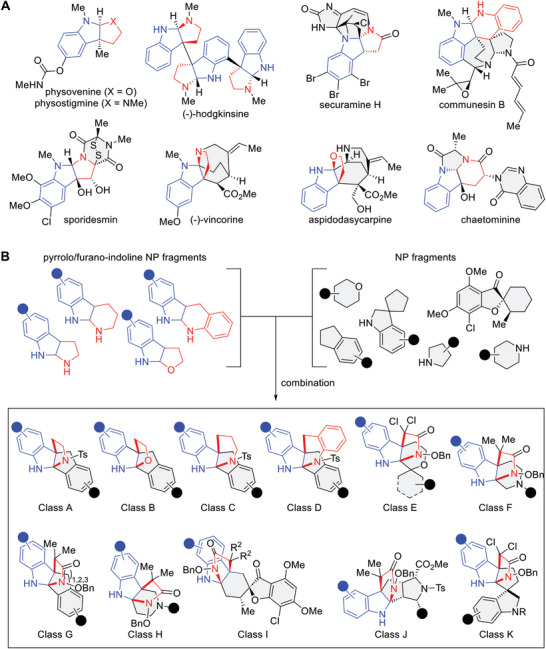
Design of the pseudo‐NP collection. A) Natural products containing 4*H*‐pyrroloindoline, 4*H*‐furanoindoline, piperidine‐indoline, or *4*H‐quinoline‐indoline fragments. B) Design of the pseudo‐natural product collection by combining 4*H*‐pyrroloindoline and related NP fragments in inset (A) with other NP fragments to afford a collection of tricyclic‐fused compounds.

Fused pyrroloindoline (PI) and furanoindoline (FI) and other related moieties are abundant in alkaloid NPs (**Figure** **1**A).^[^ ^8^ ^]^ These can be found in monomeric (such as physovenine and physostigmine) or oligomeric (hodgkinsine) forms as well as in the core scaffold fused to other NP fragments. Nature offers different fragment combinations and fusion patterns with PI and FI fragments that give rise to a diverse range of bioactivities. With this in mind, we employed the pseudo‐NP design concept to combine PI, FI, or closely related fragments with other NP fragments to afford combinations that are not found in Nature to explore biologically relevant NP‐like chemical space (Figure 1B).

## 2 Results

### 2.1 Design and Synthesis of a Pseudo‐Natural Product Collection

**Figure 2:**
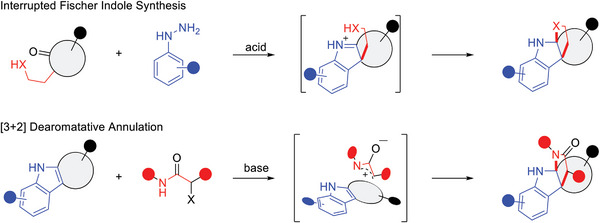
Synthetic strategies to access tricyclic‐fused pseudo‐natural products. The gray circles represent natural product fragments.

To ensure the exploration of three‐dimensional space, the NP fragments were envisioned to be combined via tricyclic core connectivity with three rings linked by means of an edge fusion pattern. Small molecules with highly three‐dimensional structures are underrepresented in many screening collections and may be less promiscuous and more likely to modulate proteins that have been previously difficult to target.^[^ ^9^ ^]^ Two synthetic strategies employing complexity‐generating reactions were envisioned to efficiently construct various fused tricyclic scaffolds (**Figure** **2**). The first strategy employs an interrupted Fischer indole (iFI) reaction^[^ ^8^, ^10^ ^]^ in which cyclic ketone NP fragments bearing an α‐tertiary carbon equipped with a nucleophilic tether could undergo a typical Fischer indole reaction sequence. This strategic design of ketone substrates would prevent aromatization, resulting in an indolenine intermediate that can react with a nucleophilic tether to construct a fused tricyclic core. Depending on the design of the tether, different ring systems can be accessed such as pyrrolidine, 4*H*‐furan, piperidine, and 4*H*‐quinoline. The second strategy involves using indole substrates that are fused to a second NP fragment. These relatively planar compounds could be dearomatized^[^ ^11^ ^]^ via [3+2] cycloadditions^[^ ^12^ ^]^ to construct the pyrrolidine moiety and afford the desired 3D fused tricyclic scaffolds.

**Figure 3:**
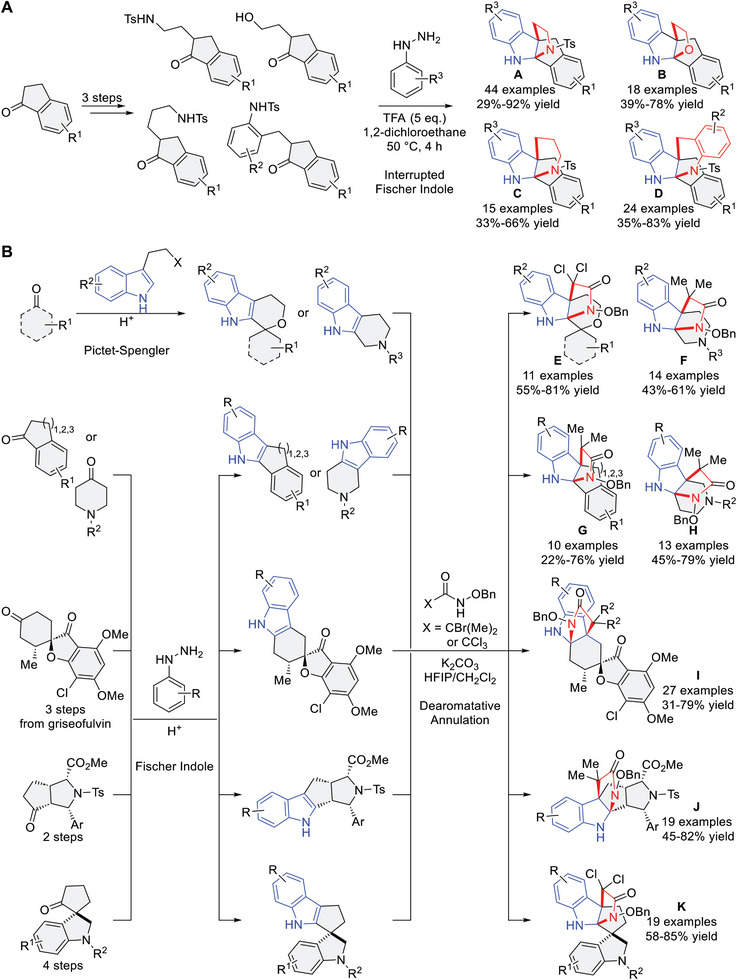
Synthesis of the pseudo‐NP compound collection. Different strategies employing A) interrupted Fischer‐indole reactions or B) [3+2] dearomatative annulations. Isolated yields for the final step of the syntheses are shown. For more synthetic details of iFI and indole dearomatization substrates, see Figures S1–S3 (Supporting Information). HFIP = 1,1,1,3,3,3‐hexafluoroisopropanol.

Following these synthetic strategies, 11 compound classes were designed and synthesized (**Figure** **3**). Classes A‐D combined fused indoline fragments with indane fragments via iFI reactions as the key step (Figure 3A). Over three synthetic steps, various indanone derivatives could be α‐functionalized to include a nucleophilic arm (Figure S1, Supporting Information). Under optimized conditions (Table S1, Supporting Information), the α‐functionalized indanone derivatives readily reacted with various aryl hydrazines to afford the desired iFI products (Classes A‐D) in moderate to high yields. Classes A‐D all contain indane NP fragments but differ in their second NP fragment due to variation in the nucleophilic arm. This resulted in the second fragment of compound classes being either a 4*H*‐pyrroloindoline (A), 4*H*‐furanoindoline (B), piperidine‐indoline (C), or 4*H*‐quinoline‐indoline (D), respectively.

Compound classes E‐K were constructed by employing dearomative indole annulation as the key step (Figure 3B). Ketones are useful functional groups for installing NP fragments.^[^ ^13^ ^]^ In this case, several NP fragments containing ketones that vary in size, complexity, and heteroatom content were synthesized in less than five steps (Figures S2 and S3, Supporting Information). These ketones were employed as substrates in either Pictet‐Spengler or Fischer Indole reactions to install indole fragments in a single step. These various indoles were then subjected to dearomative annulation conditions by employing α‐haloamides in the presence of K_2_CO_3_ in a mixture of 1,1,1,3,3,3‐hexafluoroisopropanol/CH_2_Cl_2_.^[^ ^12^ ^]^ The reaction proved to be robust and was applicable to all of the indole substrates, resulting in the desired tricyclic‐fused products in moderate to excellent yields for each class. All of these compound classes contain a 4*H*‐pyrroloindoline fragment in combination with either 4*H*‐pyran (Class E), piperidine in different orientations (Class F and Class H), indane (Class G), tetralin (Class G), benzosuberane (Class G), griseofulvin (Class I), pyrrolidine‐derived (Class J), or spiroindimicin (Class K) fragments.

**Figure 4:**
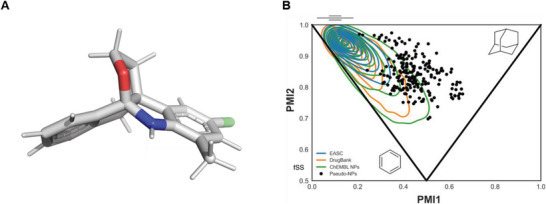
3D structure representation and principle moment of inertia (PMI) plot of the pseudo‐NPs and reference sets. A) 3D structure representation of compound **B12**. B) PMI plot. The compound sets are represented as following: tricyclic fused Pseudo‐NPs (red circles) and Enamine Advanced Screening Collection (EASC, blue lines), DrugBank compounds (DrugBank, orange lines), and bioactive NPs found in the ChEMBL database (ChEMBL NP, green lines). The densities of the reference compound sets are shown in a contour format. Different areas of the plot are indicative of different shapes (top‐left = rod‐like, bottom‐middle = disk‐like, top‐right = sphere‐like). The contour lines represent a Gaussian kernel density estimation with 10 steps. Number of reference compounds in each set: EASC = 527411 (50000 random compounds were selected), DrugBank = 4866, ChEMBL NPs = 45679.

In total, 214 pseudo‐NPs were synthesized encompassing 11 different compound classes. The 3D structures of Classes A‐E and Classes G‐K were unambiguously confirmed by single‐crystal X‐ray analyses (see the “X‐ray analyses” section in the Supporting Information).^[^ ^14^ ^]^ Each class has a tricyclic core connectivity with three rings linked by means of an edge fusion but varies in NP fragment combination or fragment orientation (see **Figure** **4**A). To ensure novelty of the pseudo‐NP compound classes relative to NPs, fragment combination and scaffold searches were conducted in the Dictionary of Natural Products (Figure S4, Supporting Information). The precise combinations that were made, i.e., scaffolds, do not occur in NPs; however, in rare cases, the three NP fragments occur in NPs but with different connectivities and in different arrangements.

### 2.2 Shape Analysis of the Pseudo‐Natural Product Collection

The three‐ring edge fusion core was chosen to ensure three‐dimensionality in the resulting scaffolds. To assess the shapes within the pseudo‐NP collection, a principle moments of inertia (PMI) plot was generated (Figure 4B).^[^ ^15^ ^]^ For comparison, ChEMBL NPs (representing bioactive NPs),^[^ ^16^ ^]^ DrugBank compounds (representing approved and investigational drugs),^[^ ^17^ ^]^ and Enamine Advanced Screening Collection (EASC, representing a synthetic screening collection)^[^ ^18^ ^]^ were used as reference sets. Relative to the three reference sets, the pseudo‐NPs are significantly shifted away from the rod/disk‐like axis and heavily populate more spherical/three‐dimensional shapes.

### 2.3 Biological Evaluation of the Pseudo‐NP Collection

The unprecedented combination of NP fragments in arrangements not found in Nature has led to the construction of novel scaffolds that are not yet linked to bioactivity. To broadly evaluate the biological activities of the pseudo‐NP collection, the cell painting assay (CPA) was employed.^[^ ^19^ ^]^ The CPA is a morphological profiling assay that quantifies phenotypic changes in cells upon compound treatment by employing six fluorescent dyes that selectively stain various cellular organelles and components, i.e., mitochondria, endoplasmic reticulum, DNA, Golgi, actin cytoskeleton, RNA, and nucleoli. High‐content fluorescence microscopy using five different channels and subsequent image processing using Cellprofiler^[^ ^20^ ^]^ followed by in‐house developed downstream processing can extract 579 highly reproducible features. Normalized to DMSO controls, these features make up a phenotypic profile of a compound that describes the cells’ morphological changes upon compound treatment.

For measuring activity in the CPA, induction values can be calculated which are the percentage of features that are significantly changed, i.e., absolute Z‐score value >3, relative to DMSO controls.^[^ ^21^ ^]^ Compounds that have induction values ≥5% are considered active. The collection was found to be enriched in morphological activity, as 57% of compounds were active at 10 µm, and all classes had active compounds (Figure S5, Supporting Information). In comparison, it was observed that only 35% of reference compounds with annotated bioactivities (1559/4504) and 32% of previously investigated internal compounds (4720/14634) are active at up to 10 µm. At concentrations up to 50 µm, 85% of the pseudo‐NPs were active.

Individual CPA profiles of active compounds can be directly compared to profiles recorded for reference compounds with annotated bioactivities, i.e., compounds with known targets or mode of action (MoA), to generate a target or mode of action hypothesis.^[^ ^22^ ^]^ Profile similarity is described by biosimilarity (BioSim), which is based on Pearson‘s correlations, and profiles are considered biosimilar for BioSim ≥75%. As profile similarities for reference compounds frequently are not related to their annotated targets, we have so far defined 12 different bioactivity clusters that are based on profile similarity, i.e., AKT/PI3K/MTOR, Aurora kinases, BET, DNA synthesis, HDAC, HSP90, lysosomotropism/cholesterol homeostasis, Na^+^/K^+^ ATPases, protein synthesis, pyrimidine synthesis, tubulin and uncoupling of the mitochondrial proton gradient.^[^ ^23^ ^]^

**Figure 5:**
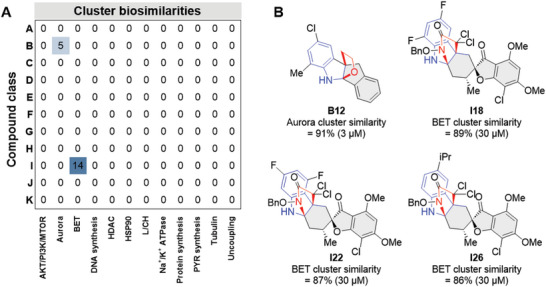
CPA subprofile analysis suggests Aurora kinase‐ and BET‐targeting compounds. A) Subprofile analysis of the pseudo‐NP collection. The values are the percentage of compounds in each compound class that have ≥85% similarity to a bioactivity cluster profile. B) Structures of compounds that have ≥85% similarity to a bioactivity cluster profile. For a subprofile analysis of **B12**, **I18**, **I22**, and **I26** at different concentrations, see Figure S6 (Supporting Information).

To facilitate target hypothesis generation, we recently introduced the concept of subprofile analysis that extracts similar features for a set of biosimilar profiles. These subprofiles are then employed for the calculation of a median profile for each bioactivity cluster and allow rapid assessment of biosimilarity of profiles to the 12 different clusters.^[^ ^23^ ^]^ Compounds that have a BioSim ≥85% to a bioactivity cluster subprofile are considered similar. A subprofile analysis was conducted for all CPA‐active profiles, i.e., with induction ≥5%, of the pseudo‐NP collection with minimum relative cell counts >50% at concentrations ranging from 1–50 µm. Gratifyingly, four compounds from various subclasses were characterized by subprofiles that had high similarities (≥85%) to either Aurora kinase (**B12**) or bromodomain and extra‐terminal motif protein (BET; **I18**, **I22**, **I26**) bioactivity cluster profiles (**Figure** **5**).

**Figure 6:**
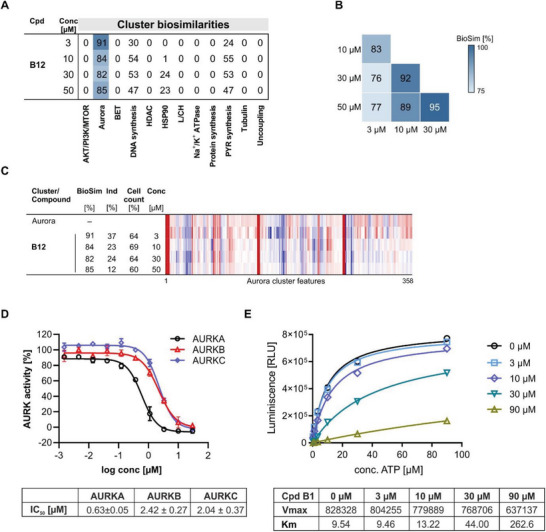
CPA reveals Aurora kinase inhibition by compound **B12**. A) Cluster biosimilarity heatmap for **B12**. PYR: pyrimidine; L/CH: lysosomotropism/cholesterol homeostasis. Values are % biosimilarity. B) Cross‐similarity for the full profiles of **B12** at different concentrations. C) Profile similarity for **B12** to the Aurora cluster profile. The Aurora cluster profile is set as a reference profile to which the following subprofiles are compared. Blue color: decreased feature, red color: increased feature. BioSim: biosimilarity, Ind: induction, Conc: concentration. D) Inhibition of Aurora kinases by **B12**. Plotted data (N = 2) are representative of two independent experiments (n = 2). IC_50_ values are mean values of N = 2, n = 2 ± SD. E) Michaelis–Menten kinetics for Aurora B inhibition by **B12**. Data are representative of n = 2. See also Figure S7 (Supporting Information).

For further validation of the bioactivity cluster predictions of the CPA, we selected the putative Aurora kinase targeting pseudo‐NPs. Class I compounds with high similarities to the BET cluster profile were not investigated further as BET‐similar profiles occurred only at high concentrations, i.e., ≥30 µm. Subprofile analysis suggested modulation of Aurora kinases for **B12** as the similarity to the Aurora cluster was 91% and 84% at 3 and 10 µm, respectively (**Figure** **6**A–C). Increasing the concentration to 30 or 50 µm led to similar results (Figure 6A). The full profiles of **B12** were compared and are biosimilar to each other (Figure 6B), indicating a similar phenotype induced at these different concentrations. Aurora kinases are mitotic kinases with highly conserved C‐terminal kinase domain between the family members.^[^ ^24^ ^]^ Although Aurora kinases could rescue each other, their variable N‐terminal domains mediate interaction with proteins thereby resulting in specific cellular localization that most likely determines the role of each family member during cell division.^[^ ^24^ ^]^ Aurora A is involved in centrosome maturation and bipolar spindle assembly, whereas Aurora B and C regulate condensation, orientation, and attachment to chromosomes to kinetochores during pro‐meta phase and for cytokinesis.^[^ ^24^ ^]^

Compound **B12** decreased the cell count to ≈65% at 3–50 µm (Figure 6C), which was also observed for Aurora kinase inhibitors that define the Aurora cluster in CPA (Table S2, Supporting Information). It should be noted that cell counts > 50% do not necessarily represent reduced cell viability and may only be indicative of cell cycle arrest as there is a short incubation period of 20 h in the CPA. Analysis of Aurora kinase activity in vitro upon treatment with **B12** revealed inhibition of all three Aurora kinases with IC_50_ values of 597 nm, 2.2 µm, and 2.3 µm for Aurora A, Aurora B, and Aurora C, respectively (Figure 6D). To establish a structure‐activity relationship, the remaining compounds from Class B and selected compounds from Classes A, C, and D were similarly evaluated at 10 µm. None of the 24 derivatives inhibited Aurora A, B, or C in vitro which suggests a narrow structure‐activity relationship (Table S3, Supporting Information). These results are in line with the structure‐bioactivity relationship based on the CPA data that did not detect any similarity of these derivatives to the Aurora cluster (Table S3, Supporting Information) and suggests that the CPA is a reliable tool for the detection of inhibitors of Aurora kinases.

Michaelis‐Menten kinetic studies were performed at different concentrations of ATP and compound **B12** using Aurora B. While different compound concentrations did not alter the maximal velocity (Vmax), a dose‐dependent increase in the Michaelis‐Menten constant Km was detected, indicating that **B12** is an ATP‐competitive inhibitor of Aurora B (Figure 6E). Most ATP‐competitive kinase inhibitors are flat compounds that contain aromatic heterocycles that mimic the adenine moiety of ATP.^[^ ^25^ ^]^ Compound **B12**, however, is a very unusual chemotype for an ATP‐competitive inhibitor as it has a highly three‐dimensional structure. In general, kinase inhibitors that are ATP‐competitive may lack selectivity. Indeed, a kinase panel including more than 400 wildtype (wt) kinases revealed that at 10 µM compound B12 inhibited multiple other kinases (see Table S4, Supporting Information): 19 wt kinases were inhibited by 90% and for an additional 15 wt kinases, compound **B12** interfered with the binding of an ATP‐based probe by ≥90%. **B12** did not selectively target one particular group of protein kinases^[^ ^26^ ^]^ (Table S4, Supporting Information). Several kinases that regulate Aurora kinases, mitosis or the cell cycle^[^ ^27^ ^]^ were inhibited by **B12** (Table S4, Supporting Information), which may lead or contribute to the observed CP phenotype. Therefore, we explored the CP profiles recorded for inhibitors of those kinases that were inhibited by more than 50% at 10 µm **B12**, i.e., AAK1, ACVR1, CDK1,2,7, CHK1/2, GAK, LCK, PKC mu, RSK3 and WEE1. The inhibitor PKC‐IN‐1 that targets PKC mu along with other PKC isoforms was not active in CPA. The profiles of the remaining inhibitors showed neither similarity to the Aurora cluster, nor to the profiles of **B12** at 3 or 10 µm (Table S5, Supporting Information). A similarity of 76% to the Aurora cluster was detected for the RSK inhibitor BI‐D1870 (10 µm), which is slightly below our biosimilarity threshold for cluster analysis. However, at 10 µm, BI‐D1870 almost completely also suppresses Aurora B activity,^[^ ^28^ ^]^ which explains the similarity to the Aurora cluster. Hence, although **B12** has a moderate kinase selectivity in vitro, its morphological profile in U‐2OS cells at 10 µm is linked to inhibition of Aurora kinases.

**Figure 7:**
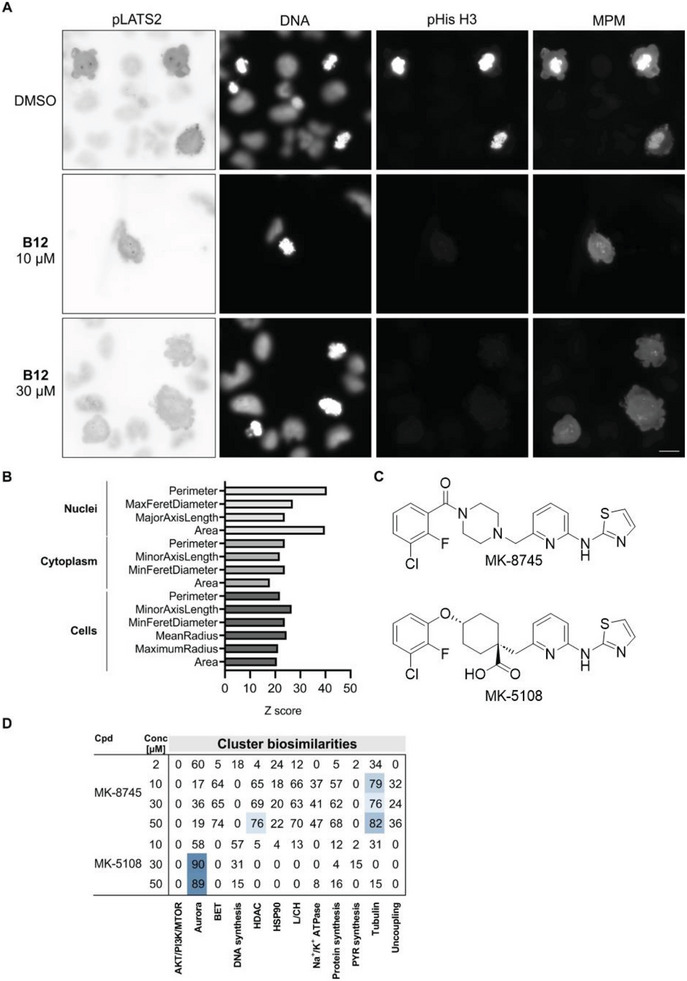
Influence of **B12** on Aurora kinase activity in cells. A) Detection of pLATS and phospho‐Histone H3 (pHis H3) as a marker of Aurora A or Aurora B activity, respectively. U‐2OS cells were treated with the compound for 8 h prior to staining for pLATS and pHis H3 and for the mitotic phosphopeptides using an MPM antibody. Inverted images are shown for pLATS2. Scale bar: 20 µm. B) Z scores for altered features related to area shape for compound **B12** (10 µm) as determined in CPA. C) structures of the selective Aurora A inhibitors MK‐8745 and MK‐5108. D) Cluster biosimilarity heatmap for MK‐8745 to MK‐5108. PYR: pyrimidine; L/CH: lysosomotropism/cholesterol homeostasis. Values are % biosimilarity.Conc: concentration. See also Figures S8 and S9 (Supporting Information).

The Aurora cluster‐defining compounds exhibit different selectivities for the three Aurora family members (Table S6, Supporting Information). As Aurora kinases exert specific functions during cell division, the phenotype detected by CPA may be due to the modulation of one particular Aurora kinase or, alternatively, the morphological changes may be due to a mixed phenotype, i.e., inhibition of two or three Aurora kinases. In U‐2OS cells, Aurora C is expressed only in germ cells and Aurora C mRNA in U‐2OS cells are 20% of Aurora B level.^[^ ^29^ ^]^ As suggested by de Groot et al., predominantly the activity of Aurora A and B may be detected in U‐2OS cells and, therefore, we explored the phosphorylation of LATS2 (Ser83) and histone H3 (Ser 28) as substrates of Aurora A and Aurora B, respectively.^[^ ^29^ ^]^ Cells were treated for 8 h with the compound prior to detection of mitotic cells using an MPM antibody, which stains mitotic phosphopeptides. Treatment of U‐2OS cells with 1 µm **B12** did not impair phosphorylation of LATS2 or His H3 (**Figure** **7**A). However, at 10 and 30 µm, phosphorylation of Histone H3 (Ser 28) was completely abolished, whereas pLATS2 (Ser 83) was still detectable at 10 µm and was reduced at 30 µm (Figure 7A).

These findings indicate that **B12** primarily impairs Aurora B activity in U‐2OS cells. Inhibition of Aurora kinases by small molecules leads to increase in cell size.^[^ ^30^ ^]^ Upon treatment with **B12**, substantially higher Z scores were observed in the CPA for features related to the shape of nuclei, cytoplasm and the cells, in general (Figure 7B; Table S7, Supporting Information). Large‐nuclei phenotype can be caused by Aurora B inhibitors with cells displaying irregular nuclear shape as a result of polyploidization.^[^ ^31^ ^]^ Indeed, in presence of **B12** cells exhibited nuclei with irregular shape (see Figure S8, Supporting Information) further confirming inhibition of Aurora B in cells.

We analyzed the profiles of Aurora inhibitors in the CPA in more detail. The Aurora cluster is defined by compounds that inhibit both Aurora A and B, like ZM‐447439, danusertib, AT9283, SNS‐314, AMG 900, CCT 137690, ENMD‐2076 L‐(+)‐tartaric acid, or selective Aurora B inhibitors such as barasertib and GSK‐1070916 (Table S6, Supporting Information). According to de Groot et al., only MK‐8745 and MK‐5108 achieve specific Aurora A inhibition in cells, whereas many other inhibitors primarily target Aurora B in cells.^[^ ^29^ ^]^ No profile cross‐similarity was detected for the profiles of MK‐8745 and MK‐5108 (Figure S9, Supporting Information). For the profiles of MK8745, no similarity to the Aurora cluster was detected, whereas at higher concentrations, similarity to the tubulin cluster became apparent (Figure 7D). The profiles of MK‐5108 at 30 and 50 µm displayed similarity to the Aurora cluster (Figure 7D). However, we could not detect any profile biosimilarity for MK‐8745 and MK‐5108 at lower concentrations that may stem from an Aurora A‐specific phenotype (Figure S9, Supporting Information) despite the high potency of the compounds (IC_50_ values for Aurora A inhibition for MK‐8745: 0.6 nm; IC50 (MK‐5108): 0.064 nm).^[^ ^32^ ^]^ Therefore, it remains elusive whether inhibition of Aurora A can be detected at the conditions used for CPA (i.e., compound treatment for 20 h in U‐2OS cells). Along these lines, Marugàn et al. reported that 48 h are required to detect Aurora A‐related phenotypes in cells.^[^ ^33^ ^]^ Our findings suggest that the Aurora cluster profile is most likely related to inhibition of Aurora B. Of note, inhibition of Aurora C, which is expressed in U‐2OS cells at lower levels than Aurora B,^[^ ^29^ ^]^ cannot be excluded.

### 2.4 Molecular Docking to Reveal the Binding Mode of B12 in the ATP‐Binding Site of Aurora Kinases

Both enantiomers of **B12** were docked into three Aurora A structures (PDB ID 5EW9, 3H10, and 3FDN) each displaying differences in activation loop (A‐loop), ATP‐binding loop (P‐loop), and DFG‐motif conformations. Structures 5EW9 and 3H10 display a more open P‐loop and closed A‐loop configuration that provides an enclosed and more “shapely” active site pocket, while 3FDN is less enclosed and open to solvent space.

**Figure 8:**
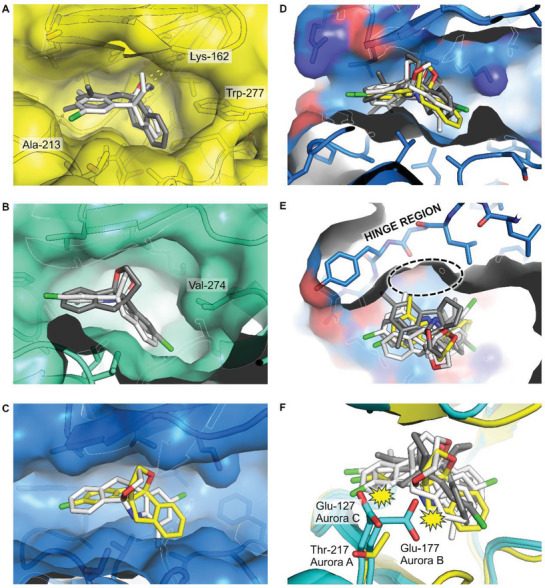
Molecular docking of **B12** into the Aurora A kinase domain. A). **B12** Enantiomers 1 and 2 docked into PDB structure 5EW9. Ent‐1 docking (grey) gives 7/10 solutions closely overlaid. This pose has two weak hydrogen bonds (dashed lines), one ring oxygen to Lys‐162, and one halide bond to Ala‐213 NH. Ent‐2 docking (white) also gives 7/10 solutions closely overlaid with a possible, weak hydrogen bond to Lys‐162. Both enantiomer bind through numerous non‐polar contacts including those to the Trp‐277 side chain presented from the A‐loop that closes off the binding pocket. B) **B12** Enantiomers 1 and 2 docked into PDB structure 3H10. Both Ent‐1 (grey) and Ent‐2 docking (white) give 10/10 solutions closely overlaid. The B12 molecule is again oriented in a V‐shape with the binding pocket closed off by the A‐loop similarly to 5EW9, but this time by the Val‐274 side chain. C) **B12** Enantiomer 2 docking into PDB structure 3FDN. Ent‐1 docking is not shown and affords random and improbable binding poses. Ent‐2 docking affords two sets of overlaid poses (in white and yellow) – the two poses are related by a 180‐degree rotation that maintains the V‐shaped bend. D) Combined docking poses from (A), (B), and (C). All docking poses display a V‐shape (oxygen‐containing ring up) and occupy the same location. E) The **B12** molecule could be grown to occupy space in the back of the binding pocket (dashed ellipse) and to engage with the hinge region of the kinase domain via hydrogen bonding. F) A single amino acid substitution from Thr‐217 of Aurora A to Glu‐177 or Glu‐127 of Aurora B and C, respectively, affords steric clash (yellow symbol) with the **B12** molecule that might explain the isoform selectivity observed.

Collectively across all the docked structures, the inherently bent conformation of **B12** fits into a non‐polar pocket underneath the G‐loop (**Figure** **8**A–D). The orientation of all of the docked molecules is exclusively Λ‐shaped not V‐shaped and thus the tetrahydrofuran ring is always pointed upward as shown in Figure 8. Within this constrained orientation, either enantiomer appears to bind in several different poses to occupy the same space, making extensive non‐polar contacts to the protein – very few polar contacts are evident. Docking poses in the more enclosed structures (closed or partially closed A‐loop conformation; PDB 5EW9 and 3H10) overlay more closely and suggest that **B12** may select inactive configurations of Aurora A kinase preferentially and provide some selectivity over other kinases. Additional affinity and selectivity might be achieved through chemical modification to form new polar contacts or by growing the molecule to engage with the hinge region (Figure 8E) through hydrogen bonding similarly to most other ATP‐competitive kinase inhibitors.

The docking experiments indicate a mechanism for isoform selectivity in the Aurora kinase family. The three isoforms Aurora A, B, and C are highly conserved in their kinase domain sequences and structures with most variation in loop regions not relevant to inhibitor binding in the ATP site. However, a single amino acid change from Thr‐217 of Aurora A to Glu‐177 or Glu‐127 of Aurora B and C, respectively, offers a simple isoform selectivity mechanism (Figure 8F). In this site immediately adjacent to the **B12** binding location, a glutamate side chain would sterically clash with the majority of the binding poses modeled although more subtle effects relating to protein dynamics and conformation cannot be discounted as a contributing factor for the isoform selectivity observed.

### 2.5 Cheminformatic Comparison of B12 to Established Aurora Kinase Inhibitors

**Figure 9:**
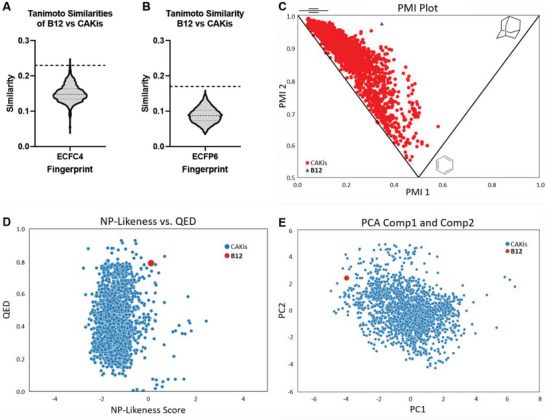
Cheminformatic analyses of pseudo‐NP **B12** to established Aurora kinase inhibitors. A) Violin plot of the Tanimoto similarities of the Morgan fingerprints (ECFC4, count fingerprint, radius 2) of ChEMBL Aurora kinase inhibitors to **B12** (range = 0.04–0.25; median = 0.15). The median and quartiles are indicated on the violin by dashed lines. The dashed line at 0.23 represents an upper threshold of randomness.^[^ ^37^ ^]^ B) Violin plot of the Tanimoto similarities of the Morgan fingerprints (ECFP6, bit fingerprint of length 1024, radius 3) of ChEMBL Aurora kinase inhibitors to **B12** (range = 0.04–0.16; median = 0.09). The median and quartiles are indicated on the violin by dashed lines. The dashed line at 0.17 represents an upper threshold of randomness.^[^ ^37^ ^]^ (C) Principle moments of inertia plot of the pseudo‐NP **B12** (blue triangle) and ChEMBL Aurora kinase inhibitors (CAKis, red circles). Different corners of the triangular plot indicate different shapes (top‐left = rod‐like, bottom‐middle = disk‐like, top‐right = sphere‐like). D) Plot of NP‐likeness scores versus Quantitative Estimation of Drug Likeness scores (QED) of the pseudo‐NP **B12** (red circle) and ChEMBL Aurora kinase inhibitors (CAKis, blue circles). E) Principal component analysis of 17 molecular descriptors (see SI for details) of the pseudo‐NP **B12** (red circle) and ChEMBL Aurora kinase inhibitors (CAKis, blue circles) showing principal components 1 versus 2. For plots of principal components 1 versus 3 and 2 versus 3, see Figure S10 (Supporting Information). Cumulated feature contribution to principal components and principal component loading can be found in Figures S11 and S12 (Supporting Information). Explained variance: PC1 = 44.1%, PC2 = 14.3%, PC3 = 11.3%. The CAKis data set is composed of 2236 compounds.

The pseudo‐NP **B12** may represent a new chemotype for Aurora kinase inhibition. Web‐based target predictions based on chemical similarity using SEA^[^ ^34^ ^]^ did not suggest any potential targets for **B12**, whereas SwissTargetPrediction^[^ ^35^ ^]^ suggested Aurora A as a putative target, albeit with a low ranking of 100th. To evaluate structural and physiochemical similarities of the pseudo‐NP to known Aurora kinase inhibitors in a broader sense, the pseudo‐NP was compared via cheminformatic methods as employed in the RDKit^[^ ^36^ ^]^ to a curated set of 2236 Aurora A, B, and/or C inhibitors that were extracted from the ChEMBL data base (for curation details, see the “Cheminformatic Details” section in the Supporting Information). Tanimoto similarities of the Morgan fingerprints (ecfc4 design) of the ChEMBL Aurora kinase inhibitors (CAKis) relative to the pseudo‐NP were calculated to determine connectivity similarities (**Figure** **9**A). The pseudo‐NP has a median similarity of 0.15 and a maximum similarity of 0.25 to the CAKis. These values align with 100 subsets containing 100 random compounds from the Enamine Advanced Screening Collection (median = 0.14, 95th percentile median = 0.23)^[^ ^37^ ^]^ and confirm the pseudo‐NP has significantly different connectivities than any of the CAKis. Similar conclusions were obtained when a fingerprint of a different design (ecfp6) was employed (Figure 9B).

Shape analysis via a principle moment of inertia (PMI)^[^ ^15^ ^]^ plot showed that the pseudo‐NP has a unique shape relative to the CAKis and is highly three‐dimensional (Figure 8C). NP‐likeness^[^ ^38^ ^]^ and quantitative estimation of drug‐likeness (QED)^[^ ^39^ ^]^ scores were calculated and plotted, and the pseudo‐NP (NP‐likeness = +0.11, QED = 0.79) was found to occupy an area that is only sparsely populated by CAKis (Figure 8D). Finally, a principal component analysis of 17 molecular descriptors^[^ ^40^ ^]^ was calculated (Figure 8E), which revealed that the pseudo‐NP occupies a unique space relative to CAKis. Taken together, these cheminformatic results indicate that the pseudo‐NP **B12** represents an unprecedented chemotype for Aurora kinase inhibition with both its structural and physicochemical properties being very distinct from established CAKis.

## 3 Conclusion

Pseudo‐NPs are the combination of NP fragments in arrangements that are not possible via known biosynthetic pathways, and their structures are therefore novel and are not yet linked to bioactivities. Herein, complexity‐generating reactions, i.e., interrupted Fischer indole and 3+2 indole dearomatization reactions, were employed to rapidly access a collection of 214 highly three‐dimensional pseudo‐NPs. Morphological profiling of the collection via the CPA coupled with a subprofile analysis facilitated the identification of a relatively highly 3D and unprecendented chemotype for Aurora kinase inhibition. Although the compound inhibits multiple kinases in vitro, predominantly an Aurora B‐related phenotype was detected in the CPA.

The pseudo‐NP concept is designed to provide compound classes that may have unexpected bioactivities and is exemplified here by the unbiased discovery of a very unusual and unique ATP‐competitive kinase inhibitor. In such early discovery efforts, it is not to be expected that initial hits are selective, and selectivity and potency need to be improved in subsequent structure‐activity campaigns. To this end, we provide an Aurora docking model which provides information for future structure optimization that may increase compound selectivity.

Future implementation of pseudo‐NP design and evaluation of the resulting collections via unbiased and biologically broad screening technologies promises facilitate the discovery of novel chemotypes with unexpected or unprecedented bioactivities.

## Conflict of Interest

The authors declare no conflict of interest.

## Supporting information

Supporting Information

Supporting Information

Supporting Information

## Data Availability

The data that support the findings of this study are available from the corresponding author upon reasonable request.
